# A new prognostic scoring system for newly diagnosed multiple myeloma in the era of new drugs

**DOI:** 10.3389/fmed.2024.1473034

**Published:** 2024-10-22

**Authors:** Ye Li, Junru Liu, Jingjing Deng, Yuan Jian, Zhiyao Zhang, Huixing Zhou, Juan Li, Wenming Chen

**Affiliations:** ^1^Department of Hematology, Myeloma Research Center of Beijing, Beijing Chaoyang Hospital, Capital Medical University, Beijing, China; ^2^Peking University People’s Hospital, Peking University Institute of Hematology, National Clinical Research Center for Hematologic Disease, Beijing Key Laboratory of Hematopoietic Stem Cell Transplantation for Hematological Diseases, Beijing, China; ^3^Department of Hematology, The First Affiliated Hospital of Sun Yat-sen University, Guangzhou, China

**Keywords:** multiple myeloma, cytogenetic abnormal, FISH, cutoff, prognosis

## Abstract

**Background:**

We developed a new predictive staging system to explore the heterogeneity of survival in newly diagnosed multiple myeloma (NDMM) patients in the real world.

**Methods:**

In this retrospective study, we evaluated the predictive value of cytogenetic abnormal and clinical data in 375 patients with NDMM at our center. Established a weighted MM prognostic scoring system risk model and validated its predicted PFS and OS by external cohort.

**Results:**

Elevated lactate dehydrogenase levels (1 point), international staging system stage II/III (1 point), 1q21+ ≥ 52.75% (0.5 point), del (17p) ≥ 3.5% (0.5 point), and t (14;16) ≥ 35.25% (1 point) had independent prognostic significance. Patients were further divided into three risk groups: low (I) (score 0–0.5, 16.5%), intermediate (II) (score 1, 46.7%), and high (III) (score 1.5–3, 36.8%). In the training cohort, the 3-year PFS was 79.5% vs. 65.3% vs. 40.3% (*p* < 0.001), and the 3-year OS was 87.7% vs.70.1% vs. 55% (*p* < 0.001) for the three risk groups. In the external validation cohort, the 3-year PFS was 85.5% vs.61.2% vs. 43.1% (*p* < 0.001) and the 3-year OS was 91.4% vs.83.5% vs. 56.9% (*p* < 0.001) for the three risk groups.

**Conclusion:**

The risk stratification of this model shows good discrimination and calibration, and its application in clinical practice can improve the risk assessment of patients with NDMM and guide personalized treatment strategies.

## Introduction

Multiple myeloma (MM) is a genetically complex and incurable disease because of the high heterogeneity of tumor biology; its clinical features, treatment responses, and outcomes are diverse (1). Accurate risk stratification is not only crucial for predicting patient prognosis accurately but also vital for guiding individualized treatment plans. Prognostic stratification models for MM have continuously evolved. Several risk stratification systems have been proposed, based on clinical features, laboratory tests, cytogenetics, and gene expression profiles. The International Myeloma Working Group (IMWG) (2) conducted a comprehensive analysis of multiple clinical studies from around the world and identified a series of independent risk factors associated with poor prognosis. Ultimately, serum albumin and β2-microglobulin levels were found to be prognostically favorable, leading to the creation of the International Staging System (ISS) (3). Owing to the absence of genetic factors in the ISS staging system, the IMWG has proposed a new staging system−Revised International Staging System (R-ISS) (4). R-ISS staging combines lactate dehydrogenase levels and cytogenetic factors high-risk chromosomal abnormalities (HRCAs) detected by interphase *fluorescence in situ hybridization* (FISH) [del(17p), t (4;14), and t (14;16)], which can more objectively and accurately reflect the prognosis of patients. The second revision of the International Staging System (R2-ISS) (5) incorporates six prognostic indicators: 1q21+ (0.5 points), t(4;14) (1 point), del(17p) (1 point), high LDH (1 point), ISS II (1 point), and ISS III (1.5 points). These indicators categorize patients into four groups, which are as follows: low-risk (I, 0 point), low-intermediate risk (II, 0.5–1 point), intermediate-high risk (III, 1.5–2.5 points), and high-risk (IV, 3–5 points). The system has better prognostic efficacy. Meanwhile, Mayo developed a prognostic stratification model for the Mayo Additive Staging System (MASS), which included high-risk IGH translocation, 1q21+, del (17p), ISS III, and elevated LDH, divided into three groups: MASS I (0 point), MASS II (1 point), and MASS III ( ≥ 2 points) (6). The latest prediction model developed by Chinese researchers, the Myeloma Prognostic Score System (MPSS) (7), is included in the elevated lactate dehydrogenase (LDH) level (1 point), ISS III (2 points), thrombocytopenia (2 points), and cumulative HRCA numbers (one HRCA 1 point, ≥ 2 HRCA 2 points), and is divided into four groups: MPSS I (22.5%, 0 points), MPSS I II (17.6%, 1 point), MPSS I III (38.6%, 2–3 points), and MPSS I IV (21.3%, 4–7 points).

However, these models all have their limitations. When using the R-ISS staging system for stratification, approximately 60% of patients are medium-risk patients, and further refinement of stratification is needed for this group of patients. Recently, 1q gain (3 copies of 1q21) or amplification ( ≥ 4 copies of 1q21), which were not included in the R-ISS, proved to be independent poor prognostic factors for newly diagnosed multiple myeloma (NDMM) (8–11). R2-ISS is a model construction aggregating global multicenter clinical trial data and cannot accurately reflect real-world situations. This study showed that although t (14; 16) had a shorter OS, but PFS was not statistically significant (HR 1.15 [95% CI, 0.96–1.37], *p* = 0.13) and therefore was not included in the calculation of R2-ISS. In MASS, a weighted prognostic system that fully reflects the main risk factors has not yet been fully established. However, the MPSS incorporates the number of high-risk abnormalities as risk factors, does not weight scores for specific abnormalities, has a cutoff value for cytogenetic abnormalities selected according to the European Myeloma Network (EMN) criteria.

Specific cytogenetic abnormalities (CAs) play a reliable prognostic role in risk stratification of patients with NDMM. In recent years, FISH has become the standard qualitative and quantitative CA analysis method for patients with MM patients (12, 13). However, the definition of the cut-off value remains controversial. The most commonly used cut-off value in clinical practice is recommended by EMN, with a critical value of 20% for abnormal chromosome count, 10% for IgH translocation, and 10% for other translocations (14). However, in the Intergroupe Francophone du Myelome (IFM) 99 test (15), the recommended critical values for del (17p) and 1q21+ were 60% and 30%, respectively. In the Mayo Clinic Institutional, the critical values of 1q21+, del(17p), and IGH translocations were 20%, 10%, and 6%, respectively (16). There are few clinical prognostic models related to the proportion of plasma cells in CA.

In this study, we attempted to develop a model of a prognostic scoring system capable of predicting PFS and OS in NDMM patients based on the clinical characteristics and genetic abnormalities of patients in a multicenter real-world setting in China, reflecting the status of real-world treatments in China, and to validate the performance and efficacy of the model using external data.

## Patients and methods

### Study population and data collection methods

We retrospectively analyzed the clinical data of all multiple myeloma patients admitted to Beijing Chaoyang Hospital affiliated with Capital Medical University from January 2018 to November 2020. After the screening, 375 NDMM patients with the complete treatment process and follow-up were collected as a training cohort. An external validation cohort was established using 189 patients with NDMM diagnosis from January 2018 to November 2020 at the First Affiliated Hospital of Sun Yat-sen University with complete medical record information and follow-up.

The baseline characteristics of the patients in the training and external validation cohorts, including age, sex, ISS stage, hemoglobin, creatinine, calcium, albumin, lactate dehydrogenase (LDH), β2-microglobulin (β2MG), and isotype, were recorded at the time of diagnosis and throughout the follow-up period. All patients were evaluated for diagnosis and treatment response using the 2014 International Myeloma Working Group (IMWG) standards.

All 564 patients received induction regimens containing at least one novel agent, including proteasome inhibitors (PIs)-based regimens, immunomodulatory drugs (IMIDs)-based regimens, or PIs combined with IMIDs regimens. Patients suitable for transplantation first undergo 4 courses of induction therapy, followed by autologous hematopoietic stem cell transplantation (ASCT) for consolidation therapy. After 3 months of ASCT, maintenance therapy such as lenalidomide should be used. Patients who are not suitable for transplantation will receive 8 cycles of induction therapy, followed by maintenance therapy with Lenalidomide or Ixazomib.

### Fluorescence in situ hybridization analysis

Before FISH, all bone marrow plasma samples were enriched using CD138-directed enrichment. FISH analysis employed a panel for the following chromosomal abnormalities: *TP53* [del (17) (p13.1)], *1q21* (1q21), *IGH/MAF* (14q32/16q23), *IGH/FGFR3* (14q32/4p16.3), and *IGH/CCND1* (14q32/11q13), excluding t (14;20), because it accounts for a lower proportion of patients with MM. A total of 200 interphase nuclei were analyzed.

### Statistical analysis

Progression-free survival (PFS) was defined as the time from the initial treatment to disease progression, recurrence, or death from any cause. Overall survival (OS) was defined as the time from initial treatment to death for any reason or the last follow-up.

Continuous variable characteristics were described using median and range. Fisher’s exact test was used to compare categorical variables among the groups, and the Mann–Whitney U nonparametric test was used for continuous variables. Survival curves were generated using the Kaplan–Meier method, and differences were tested using the log-rank test. The prognostic impact was assessed using multivariate Cox proportional risk regression analysis, reporting hazard ratios (HRs), and their 95% confidence intervals (CIs).

The time-dependent receiver operating characteristic (ROC) curves were analyzed using the survival ROC package in the R platform to determine cut-off values with the largest Youden index for PFS and OS. Cox regression models were used for univariate and multivariate analyses to identify covariates associated with PFS and OS. Co-variates with *p* < 0.15 in univariable analyses were included in multivariable analyses. Significant covariates from the Cox multivariate analysis of the training dataset were used to develop a scoring system. Weighted scores were assigned to these covariates according to the regression coefficients. The area under the receiver operating characteristic curve (AUROC) was used to estimate the accuracy of the predictive model, and calibration plots were constructed to determine how closely predicted probabilities were numerically concordant with observed outcomes (17). Calculate net benefits using the Decision Curve Analysis (DCA) model (18). Statistical analyses were performed using SPSS v29.0 (IBM, Armonk, NY, US), R version 4.3.1 (R Core Team, Vienna, Austria), The *p* < 0.05 (bilateral) is considered statistically significant.

## Result

### Clinical characteristics and treatments

In the training cohort (*n* = 375), the median follow-up period was 27 months, the median age was 62 years, and 56% of the patients were male. In the first-line treatment, 57.3% of the patients received PIs + IMiDs-based therapy, 40.8% received PIs-based therapy, and 1.9% received IMiDs-based therapy. A total of 31.2% of the patients received ASCT after induction treatment. In the external validation cohort (*n* = 189), the median follow-up was 44.3 months, the median age was 57 years, and 62.4% of the patients were male. In the first-line treatment, 11.7% of the patients received PIs + IMiDs-based therapy, 72.5% received PIs-based therapy, and 15.8% received IMiDs-based therapy. A total of 57.1% of the patients received ASCT after induction treatment. The baseline features and treatments of the training and external validation cohorts are shown in Table 1.

**TABLE 1:** Baseline features and treatments of patients in the training and external validation cohorts.

Characteristic	Total (*n* = 564)	Training dataset (*n* = 375)	Validation dataset (*n* = 189)	*p-*value
Age (year), median (range)	60 (26–85)	62 (32–85)	57(26–79)	<0.001
≥65, *n* (%)	173 (30.7)	139 (37.1)	34 (18.0)	<0.001
<65, *n* (%)	391 (69.3)	236 (62.9)	155 (82.0)	
Gender				0.149
Male, n (%)	328 (58.2)	210 (56.0)	118 (62.4)	
Female, n (%)	236 (41.7)	165 (44.0)	71 (37.6)	
ISS stage, n (%)				<0.001
1	116 (20.6)	75 (20.0)	41 (21.7)	
2	184 (32.6)	91 (24.3)	93 (49.2)	
3	264 (46.8)	209 (55.7)	55 (29.1)	
HB (g/L), median (range)	93 (29–167)	92 (29–167)	94 (47–157)	0.804
<100, *n* (%)	229 (41.6)	153 (42.4)	76 (40.2)	0.650
≥100, *n* (%)	321 (59.4)	208 (57.6)	113 (59.8)	
LDH(U/L), median (range)	171.5 (37–11411)	170 (37–1602)	177 (51–11411)	0.581
≥ULN, *n* (%)	97 (17.2)	59 (18.7)	38 (20.1)	0.237
<ULN, *n* (%)	467 (82.8)	316 (81.3)	151 (79.9)	
Calcim (mmol/L), median (range)	2.30 (1.48–33.52)	2.28 (1.48–33.52)	2.39 (1.50–3.88)	<0.001
<2.75, *n* (%)	460 (87.8)	312 (88.9)	148 (85.5)	0.402
≥2.75, *n* (%)	64 (12.2)	39 (11.1)	25 (14.5)	
β2 MG(mg/L), median (range)	5.11 (0.79–75.80)	5.84 (0.79–75.8)	4.08 (1.07–30.21)	<0.001
≥5.5, *n* (%)	223 (42.6)	159 (46.9)	64 (34.8)	<0.001
<5.5, n (%)	300 (57.4)	180 (53.1)	120 (65.1)	
Creatinine (mg/dL), median (range)	82.5 (5.0–1708)	80.9 (33.8–1436.0)	88 (5.0–1708.0)	0.057
Albumin (mg/dL), median (range)	36.65 (14.7–336)	36.2 (17.9–336.0)	34.7 (14.7–53.3)	0.018
BMPCs (%), median (range)	34 (1–98)	39 (1–98)	23.5 (1–91)	<0.001
Isotype, *n* (%)				0.392
IgG	261 (46.3)	169 (45.1)	92 (48.7)	
IgA	122(21.6)	76 (20.2)	46 (24.3)	
IgD	33 (5.9)	22 (5.9)	11 (5.8)	
IgM	1 (0.2)	1 (0.2)	0	
Light chain only	135 (23.9)	97 (25.9)	38 (20.1)	
Non-secretory	12 (2.0)	10 (2.7)	2 (1.1)	
Induction, *n* (%)				<0.001
PIs	290 (51.4)	153 (40.8)	137 (72.5)	
IMiDs	37 (6.6)	7 (1.9)	30 (15.8)	
PIs+IMiDs	237 (42.0)	215 (57.3)	22 (11.7)	
ASCT, *n* (%)				<0.001
Yes	225 (40.0)	117 (31.2)	108 (57.1)	
No	339 (60.0)	258 (68.8)	81 (42.9)	

BMPC, bone marrow plasma cells; β2 MG: serum β2-microglobulin; ASCT, autologous peripheral stem cell transplantation; ISS, international staging system; PIs, proteasome inhibitors; IMIDs, immunomodulatory drugs; LDH, lactate dehydrogenase; HB, hemoglobin.

### clone sizes of different cytogenetic aberration on impaction of prognosis

In the training cohort, we investigated the effect of a single CA distortion on the prognosis of different clone sizes. We used the survival ROC package of the R platform for time-dependent receiver operating characteristic (ROC) curve analysis to calculate the best cutoff value of PFS and OS for a single CA, with the following results: 1q21+ 52.75%, del(17p) 3.5%, t (14;16) 35.25%, t (4;14) 50%, and t (11;14) 67.5%. Based on the cutoff value calculated above, we found that patients who harbored 1q21+ and t (14;16) showed shorter PFS and OS, patients who harbored t (11;14) had shorter PFS, and patients harboring del(17p) had shorter PFS and OS. However, there was no difference between PFS and OS in patients with t (4;14) (Supplementary Figures 1, 2). The distribution of various cytogenetic abnormalities in the training and external validation cohorts is shown in Table 2.

**TABLE 2:** Primary abnormalities of patients in the training and external validation cohorts.

Cytogenetic abnormalities, *n* (%)	Total (*n* = 564)	Training dataset (*n* = 375)	Validation dataset (*n* = 189)	*p-*value
1q21+	109 (19.3)	87 (23.2)	22 (11.6)	<0.001
del(17p)	55 (9.7)	45 (12.0)	10 (5.3)	0.015
t (4;14)	63 (11.1)	54 (14.4)	9 (4.8)	0.217
t (11;14)	40 (10.7)	31 (8.3)	9 (4.8)	0.872
t (14;16)	14 (2.5)	13 (3.5)	1 (0.5)	0.450

### Univariable and Multivariate Cox analysis for survival and selection of independent prognostic factors

The individual prognostic impact of each risk factor was estimated in the training cohort. Univariate and multivariate Cox analyses of predictors of PFS and OS are listed in Supplementary Tables 1A, B. According to the results of the multivariate analysis, ISS II/III (HR = 1.719, 95% CI 1.030–2.870, *p* = 0.038; HR = 2.875, 95% CI 1.442–5.732, *p* = 0.003), elevated LDH (HR = 1.867, 95% CI 1.226–2.842, *p* = 0.004; HR = 1.784, 95% CI 1.159–2.747, *p* = 0.009), 1q21+ ≥ 52.75% (HR = 1.740, 95% CI 1.178–2.569, *p* = 0.005; HR = 1.573, 95% CI 1.047–2.363, *p* = 0.029), del(17p) ≥ 3.5% (HR = 1.959, 95% CI 1.214–3.162, *p* = 0.006; HR = 1.717, 95% CI 1.016–2.899, *p* = 0.043), t(14;16) ≥ 35.25% (HR = 3.141, 95% CI 1.507–6.548, *p* = 0.002; HR = 2.091, 95% CI 1.011–4.324, *p* = 0.047) were significant independent risk factors for shortened PFS and OS.

### Developing a predictive scoring system

According to the corresponding regression coefficients related to each risk factor in the Cox analysis of OS, assigning weighted scores to each risk factor, a weighted risk score of 1 was assigned to ISS stage II/III, elevated LDH, and t (14;16) ≥ 35.25%, while del(17p) ≥ 3.5% and 1q21+ ≥ 52.75% received a risk score of 0.5 (Table 3). By adding the risk scores together, the final grading of the predictive scoring system risk stratification was established. The predicted rating system ranges from 0 to 3 points, and as the rating increases, the outcomes of patients in the training queue gradually worsen (because there were seven patients with a score of 2 and two patients with a score of 3, these two groups of patients were combined for analysis) (Supplementary Figure 3).

**TABLE 3:** Weighted scoring and stratification of risk factors in predictive models.

Predictor	B	Score	Total score	Stage
ISS II/III	1.056	1	0–0.5	Low I (62) (16.5%)
t (14;16) ≥ 35.25%	0.738	1		
elevated LDH	0.579	1	1	Intermediate II (175) (46.7%)
del(17p) ≥ 3.5%	0.540	0.5		
1q21+ ≥ 52.75%	0.453	0.5	1.5–3	High III (138) (36.8%)

**FIGURE 1:**
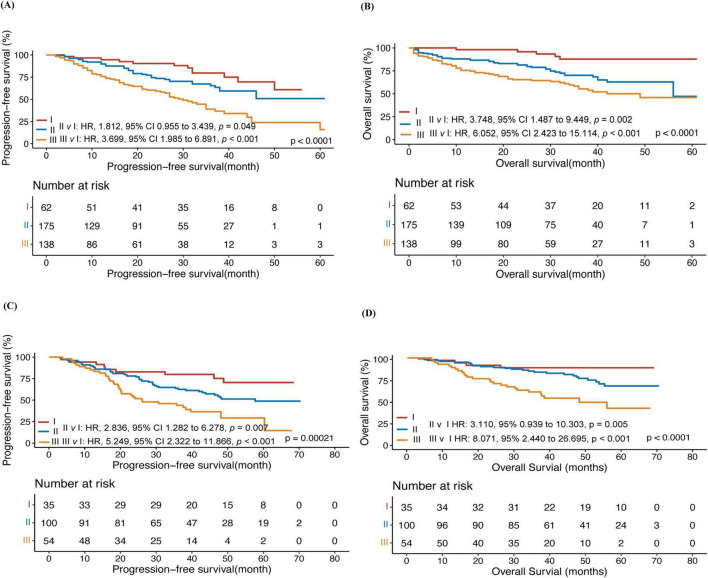
A prognostic model for predicting the prognosis of myeloma. Progression-free survival (PFS) **(A)** and overall survival (OS) **(B)** of patients in the training cohort; Progression-free survival (PFS) **(C)** and overall survival (OS) **(D)** of patients in the external validation cohort.

Patients with different scores but similar PFS and OS were combined, and the whole training cohort was segregated into three risk categories: low (I) (62) (score 0–0.5, 16.5%), intermediate (II) (175) (score 1, 46.7%), and high (III) (138) (score 1.5–3, 36.8%) (Table 3). In the training cohort, the 3-year PFS was 79.5% vs. 65.3% vs. 40.3% (*p* < 0.001), and the 3-year OS was 87.7% vs.70.1% vs. 55% (*p* < 0.001) for the three risk groups (Figures 1A, B). The differences among the different groups were statistically significant.

Then perform subgroup analysis on the new prognosis scoring system based on transplant status. Among the 258 patients who did not receive ASCT, the median PFS for the three groups of patients respectively were not reached (NR), NR, and 28 months (95% CI: 20.3–35.7), respectively (*p* = 0.00024) (Supplementary Figure 4A); the OS were NR, NR, and 38 months (95% CI: 31.3–44.7), respectively (*p* = 0.0015) (Supplementary Figure 4B). Among 117 patients undergoing ASCT, the median PFS for the three groups of patients were NR, 46 months (95% CI: 36.6–55.4), and 32 months (95% CI: 24.3–39.7), respectively (*p* = 0.0077) (Supplementary Figure 4C); and the median OS were NR, NR, and NR (*p* = 0.033), respectively (Supplementary Figure 4D).

### The external validation of the predictive scoring system

In the external validation cohort, patients were classified by the predictive model into three groups: low (I)(35) (18.5%), intermediate (II) (100) (52.9%), and high (III) (54) (28.6%). In the external validation cohort, the 3-year PFS was 85.5% vs.61.2% vs. 43.1% (*p* < 0.001) and the 3-year OS was 91.4% vs.83.5% vs. 56.9% in the low, Intermediate, and high groups, respectively (*p* < 0.001) (Figures 1C, D).

### Predictive scoring system performance

**FIGURE 2:**
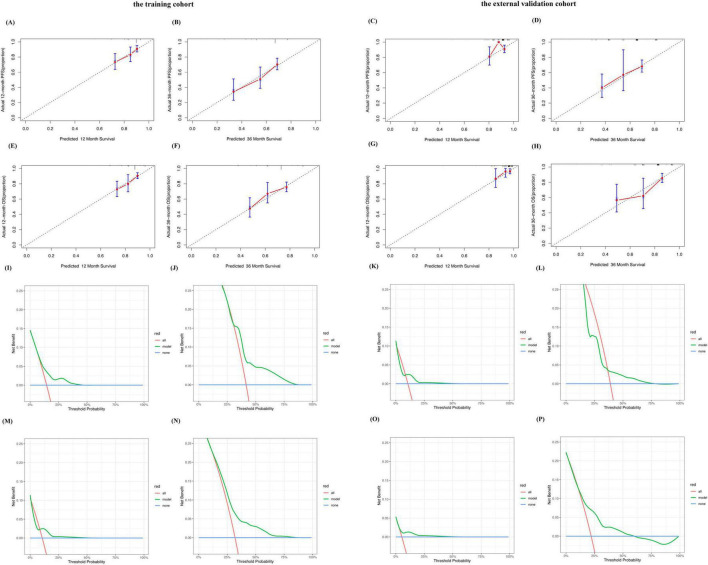
Performance of the predictive model for MM. Calibration plots of the predictive model for predicting 12-, 36-month PFS rate in the training cohort **(A,B)**. Calibration plots of the predictive model for predicting 12-, 36-month PFS rate in the external validation cohort **(C,D)**. Calibration plots of the predictive model for predicting 12-, 36-month OS rate in the training cohort **(E,F)**. Calibration plots of the predictive model for predicting 12-, 36-month PFS rate in the external validation cohort **(G,H)**. Decision curve analysis of the predictive model for predicting the probabilities of 12-, 36-month PFS in the training cohort **(I,J)**. Decision curve analysis of the predictive model for predicting the probabilities of 12-, 36-month PFS in the external validation cohort **(K,L)**. Decision curve analysis of the predictive model for predicting the probabilities of 12-, 36-month OS in the training cohort **(M,N)**. Decision curve analysis of the predictive model for predicting the probabilities of 12-, 36-monthOS in the external validation cohort **(O,P)**.

Bootstrap resampling was used to plot calibration curves for 12 month and 36 month PFS, and OS rates after treatment. The results showed that in the training cohort, the correlation between the prediction and actual outcomes was the best (Figures 2A, B, E, F). The 12 month and 36 month calibration curves of the external validation cohort showed similar excellent correlations (Figures 2C, D, G, H). These results confirmed the effectiveness and practicality of the model in an independent validation cohort. The DCA curve showed that the model had good net benefits in clinical applications, both in the training set (Figures 2I, J, M, N) and external validation sets (Figures 2K, L, O, P).

We plotted ROC curves for PFS and OS at 12 and 36 months in the training and external validation cohorts (Supplementary Figures 5A–H). This model showed good sensitivity and specificity.

### Comparison of predictive scoring system with R2-ISS

**FIGURE 3:**
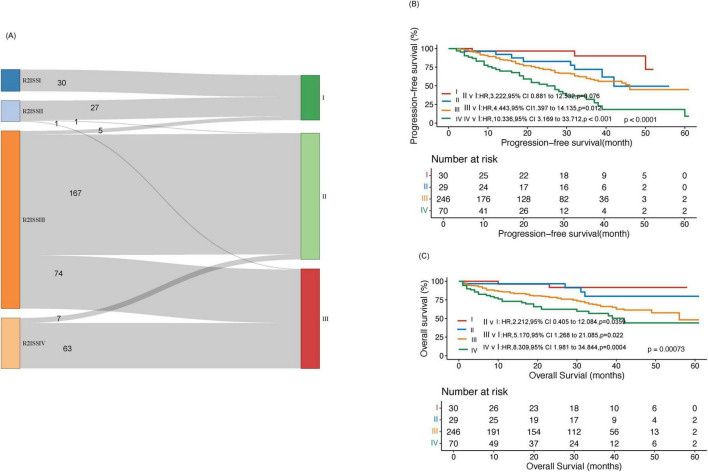
Comparison between the new prognostic scoring system and R2-ISS. **(A)** Reassignment from R2-ISS to the new prognostic scoring system in the training cohort. **(B)** Progression-free survival (PFS) analysis in the training cohort within R2-ISS. **(C)** Overall survival (OS) analysis in the training cohort within R2-ISS.

We also described the redistribution of patients from R2-ISS to the new prognostic scoring system (Figure 3A). Figure 3A shows that there were 5 (2.0%) cases of 246 R2-ISS Stage III patients transferred to Stage I in the new scoring system, 167 cases (67.9%) transferred to Stage II, and 74 cases (30.1%) assigned to Stage III in the new scoring system. The difference in PFS of newly classified patients is statistically significant (*p* = 0.026, Supplementary Figure 6). At the same time, we compared the survival of our cases in R2-ISS. The results showed that among the 375 patients, there were 30 patients (8%) in R2-ISS Stage I, 29(15.7%) in R2-ISS Stage II, 246(65.6%) in R2-ISS Stage III, and 70(18.7%) in R2-ISS Stage IV. The median PFS for these four groups of patients were as follows: NR, 42 months, 46 months (95% CI 37.3–54.7) and 25 months (95% CI 17.0–33.0) (*p* < 0.0001) (Figure 3B). The median OS for these four groups of patients were NR, NR, 56 months, and 42 months (95% CI 30.0–54.1) (*p* = 0.00073) (Figure 3C).

## Discussion

This study utilized real-world clinical data from multiple centers in China, integrating ISS staging, LDH levels, and FISH detection of CA [del (17p), t(14;16), 1q21+] as five variables of clear prognostic significance for patients, constructing a novel predictive scoring system. The scoring system classifies patients into three risk categories. Compared to the current clinical staging systems R-ISS, R2-ISS, MASS, and MPSS, this new prognostic system possesses its unique characteristics. Cox multivariate analysis results showed that Elevated LDH, ISS II/III, 1q21+ ≥ 52.75%, del (17p) ≥ 3.5%, and t (14;16) ≥ 35.25% were independent risk factors affecting PFS and OS.

The ISS is considered the first simple and reliable MM hazard stratification. The ISS staging system has broad clinical applications. However, it only includes serum albumin and β2MG levels, excluding cytogenetics-related factors (3). So when stratifying the risk of MM, researchers often analyze ISS and other risk factors together. Serum LDH is an important biomarker of MM and is frequently associated with poor prognosis in MM (19). Elevated LDH significantly affects the survival of patients, and currently, multiple prognostic assessment systems also include it as a risk factor (5–7, 20, 21).

We are trying to explore whether the percentage of each CA in FISH testing affects survival. Therefore, we calculated the cutoff values of each CA in FISH testing based on the survival status of patients. Some studies found that different proportions of CA can affect the prognosis of MM patients (11, 22). The optimal cut-off value for FISH detection of abnormal cytology remains a controversial issue that may affect prognosis and further treatment (11, 15, 16, 20, 22). Luo et al. (20) have explored the development of new predictive models based on the proportion of different CAs. However, this immature model has not been subjected to hazard stratification and performance validation. The CA threshold values in R2-ISS were defined based on the thresholds of each laboratory, whereas the cutoff values for CA in MASS were 1q21 20%, del (17p) 10%, and IGH translocation 6%, which did not show survival at this threshold, the critical value of cytogenetic abnormalities selected by MPSS based on the EMN criteria. These prognostic models did not further explore the actual significance of the percentage of cytogenetic abnormalities on survival. We utilized survival data from 375 patients at our center to analyze the prognostic significance of various abnormal percentages in FISH. The results showed that the cutoff values for each FISH CA were as follows: del (17p) 3.5%, 1q21+ 52.75%, t (14;16) 35.25%, t (4;14) 50%, and t (11;14) 67.5%.

T (14;16) is also considered a high-risk factor in mSMART risk stratification (23) and has been incorporated into the R-ISS. It is a rare translocation that accounts for approximately 3.5% of MM, mainly involving IGH sites and oncogene c-MAF, resistance to proteasome inhibitors is developed through overexpression of c-MAF (24). However, due to the rarity of t (14;16) chromosomal abnormalities and their often concurrent occurrence, their poor prognostic significance has been questioned (25, 26). In addition, R2-ISS considered t (14;16) to be significantly significant for OS but not for PFS, therefore it was not included. We calculated the cutoff value of t (14;16) using survival as 35.25%, with 13 cases in the training group (3.5%) and 2 cases in the external validation group (1.0%). The training cohort showed a statistically significant difference in PFS and OS between the t (14;16) and non-t (14;16) groups. Cox multivariate analysis showed that, regardless of PFS or OS, t (14; 16) ≥ 35.25% had independent prognostic significance. Therefore, t(14;16) can still serve as a high-risk cytogenetic abnormality in multiple myeloma (MM) and potentially holds therapeutic significance (27).

Similar to the R2-ISS and MASS, 1q21 was included as an independent prognostic risk factor in our prognostic scoring system. Research has shown that 1q21 is considered a poor prognostic marker in MM (8, 28). It has also been incorporated into prognostic models in some studies (5, 7, 20). Studies have also explored the impact of the percentage of 1q21+ on prognosis, with cut-off values ranging from 5 to 39% for 1q21 (11, 15, 16, 20, 22). In our study, both PFS and OS were meaningful when the ratio of 1q21+ plasma cells was ≥ 52.75%. Recent studies have found that the copy number of 1q21 is associated with the prognosis of patients with MM. Patients with 1q21 amplification ( ≥ 4 copies) have an extremely poor prognosis (11, 29), and the impact of 1q21 copy number on prognosis should also be considered in future prognosis evaluations.

The definition of t(4;14) in the revised International Staging System (R-ISS) is one of the HRCA. MASS and MPSS included t (4;14) as a risk factor for high-risk IGH translocation, while R2-ISS also included t (4;14) as a separate prognostic risk factor in the model. In the era of new drugs, multiple studies have shown that the negative impact of t(4;14) has been alleviated to some extent (30, 31). In recent years, some studies have raised doubts about the high-risk prognosis of t (4; 14), they have shown that t(4;14) is not a high-risk abnormality, and only those with other high-risk abnormalities showed poor survival (32). In our model, the cut-off value for t(4;14) is 50%, and t(4;14) ≥ 50% is not a prognostic risk factor with significance for both OS and PFS. Among 375 patients in the training cohort, 98.3% received a chemotherapy regimen containing bortezomib. The improvement in survival in patients with t (4;14) may be related to the higher proportion of bortezomib use.

In R-ISS, about 60% of patients are categorized as intermediate-risk (R-ISS II), and the low- or high-risk population is usually too small, resulting in uneven stratification and some high-risk patients cannot be better identified. In our model, 53.3% of NDMM patients are in stage I (16.5%) and stage III (36.8%), while 46.7% of patients are in stage II. So compared to R-ISS staging our model has a better balance of prognostic stratification.

Compared with several new prognostic scoring systems, such as R2-ISS, and MASS, the characteristics of our developed model include the use of clinical data from multiple centers in China to develop and validate it, which can truly and comprehensively reflect the treatment status in China. R2-ISS used data from a global multicenter clinical trial, and the utility of the model needs to be further validated in the real world. Our model focuses on studying the prognostic value of the percentages of various FISH CAs for MM. The risk-weighted value of the model was obtained based on the risk coefficients of five risk factors, rather than using the number of risk factors for risk stratification, such as MASS, which further reflects the true risk value. In our model, it can be seen that the risk coefficients for each type of CA are not the same, indicating that the prognostic value of each type of CA is not the same. Therefore, compared with the scoring system based on the number of high-risk CAs in MPSS, it is more convincing.

When analyzing the subgroups of patients who underwent ASCT and those who did not, this new predictive scoring system still demonstrated practical utility. However, due to the relatively small sample size of our study cohort, the low proportion of patients receiving ASCT, and the insufficient follow-up duration, the predictions regarding patient survival were not optimal. Future research should aim for larger sample sizes and longer follow-up periods to more accurately assess the impact of treatment on survival. Additionally, we evaluated this new prognostic scoring system using clinical decision curves (DCA), the Area Under the Receiver Operating Characteristic Curve (AUROC), and calibration plots, with results indicating that the predictive model may have a certain clinical relevance in terms of its benefits for clinical decision-making, predictive ability, and reliability.

Our study has some limitations. Firstly, the relatively small number of cases included in this study (comprising both the training cohort and the external validation cohort) and the relatively short follow-up period may have a certain impact on the performance of our model. In addition, we did not evaluate some cytogenetic abnormalities, such as the del(1p32), that could not be obtained. We also did not consider the impact of the 1q21+ copy number on prognosis. This study did not evaluate the impact of other clinical features, such as platelet count and blood calcium level, on risk stratification, and the model did not include gene mutation profiles. More cases and comprehensive data are needed to explore the prognostic stratification system of MM.

In summary, we established and validated a prognostic risk model for patients with NDMM that reflects the treatment situation in China using multicenter real-world clinical standards and cytogenetic abnormality data. Scoring-based risk stratification helps to accurately stratify the prognosis of newly diagnosed MM patients and develop more personalized treatment strategies.

## Data Availability

The original contributions presented in the study are included in the article/Supplementary material, further inquiries can be directed to the corresponding authors.
