# Supplementary material for: A new prognostic scoring system for newly diagnosed multiple myeloma in the era of new drugs
Source: Front Med (Lausanne). 2024 Oct 22;11:1473034. doi: 10.3389/fmed.2024.1473034 (PMC11536264; doi:10.3389/fmed.2024.1473034)
Supplement: Supplementary file 1 [file Data_Sheet_1.docx]

Table S1A. Univariate and multivariate Cox analysis for PFS of patients in training

cohort (*n* = 375).

|  | univariate |  |  |  |  | multivariate |
| --- | --- | --- | --- | --- | --- | --- |
| Variants | p value | HR (95% CI) |  | B | p value | HR (95% CI) |
| ISS II/III | 0.008 | 1.978(1.191-3.284) |  | 0.542 | 0.038 | 1.719(1.030-2.870) |
| t(14;16) ≥35.25% | < 0.001 | 4.188(2.025-8.660) |  | 1.145 | 0.002 | 3.141(1.507-6.548) |
| LDH high | < 0.001 | 2.198(1.457-3.317) |  | 0.624 | 0.004 | 1.867(1.226-2.842) |
| del(17p) ≥ 3.5% | 0.007 | 1.917(1.193-3.080) |  | 0.673 | 0.006 | 1.959(1.214-3.162) |
| 1q21+ ≥ 52.75% | < 0.001 | 1.915(1.308-2.802) |  | 0.554 | 0.005 | 1.740(1.178-2.569) |

Table S1B. Univariate and multivariate Cox analysis for OS of patients in training

cohort (*n* = 375).

|  |  | univariate |  |  |  | multivariate |
| --- | --- | --- | --- | --- | --- | --- |
| Variants | *p* value | HR (95% CI) |  | B | *p* value | HR (95% CI) |
| ISS II/III | < 0.001 | 3.186(1.606-6.320) |  | 1.056 | 0.003 | 2.875(1.442-5.732) |
| t(14;16) ≥35.25% | 0.005 | 2.847(1.383-5.862) |  | 0.738 | 0.047 | 2.091(1.011-4.324) |
| LDH high | 0.007 | 1.827(1.179-2.831) |  | 0.579 | 0.009 | 1.784(1.159-2.747) |
| del(17p) ≥ 3.5% | 0.079 | 1.594(0.948-2.682) |  | 0.540 | 0.043 | 1.717(1.016-2.899) |
| 1q21+ ≥ 52.75% | 0.008 | 1.730(1.157-2.587) |  | 0.453 | 0.029 | 1.573(1.047-2.363) |

Figure S1

Figure 1S. Survival analysis. PFS analysis for patients with 1q21+ ≥ 52.75% vs 1q21+ < 52.75% (A), del(17p) ≥ 3.5% vs del(17p) < 3.5% (B), t (14;16) ≥ 35.25% vs t (14;16) < 35.25% (C), t (4;14) ≥ 50% vs t (4;14) < 50% (D), t (11;14) ≥ 67.5% vs t (11;14) < 67.5% (E).

Figure S2

Figure 2S. Survival analysis. OS analysis for patients with 1q21+ ≥ 52.75% vs 1q21+ < 52.75% (A), del(17p) ≥ 3.5% vs del(17p) < 3.5% (B), t (14;16) ≥ 35.25% vs t (14;16) < 35.25% (C), t (4;14) ≥ 50% vs t (4;14) < 50% (D), t (11;14) ≥ 67.5% vs t (11;14) < 67.5% (E).

Figure S3


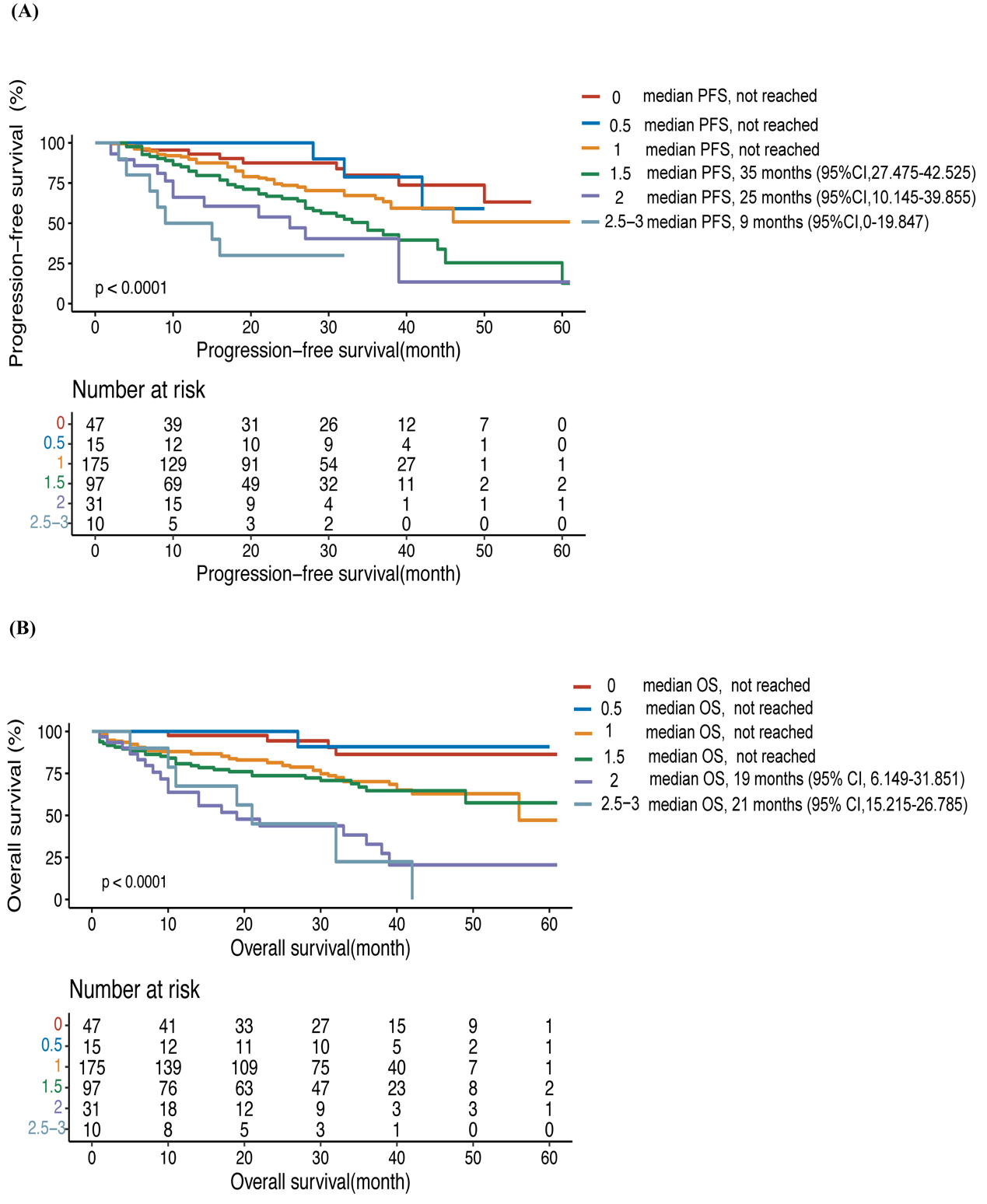


Figure S3. The prognosis for the training cohort within the prediction model score segment. PFS (A) and OS (B) of different scores.

Figure S4


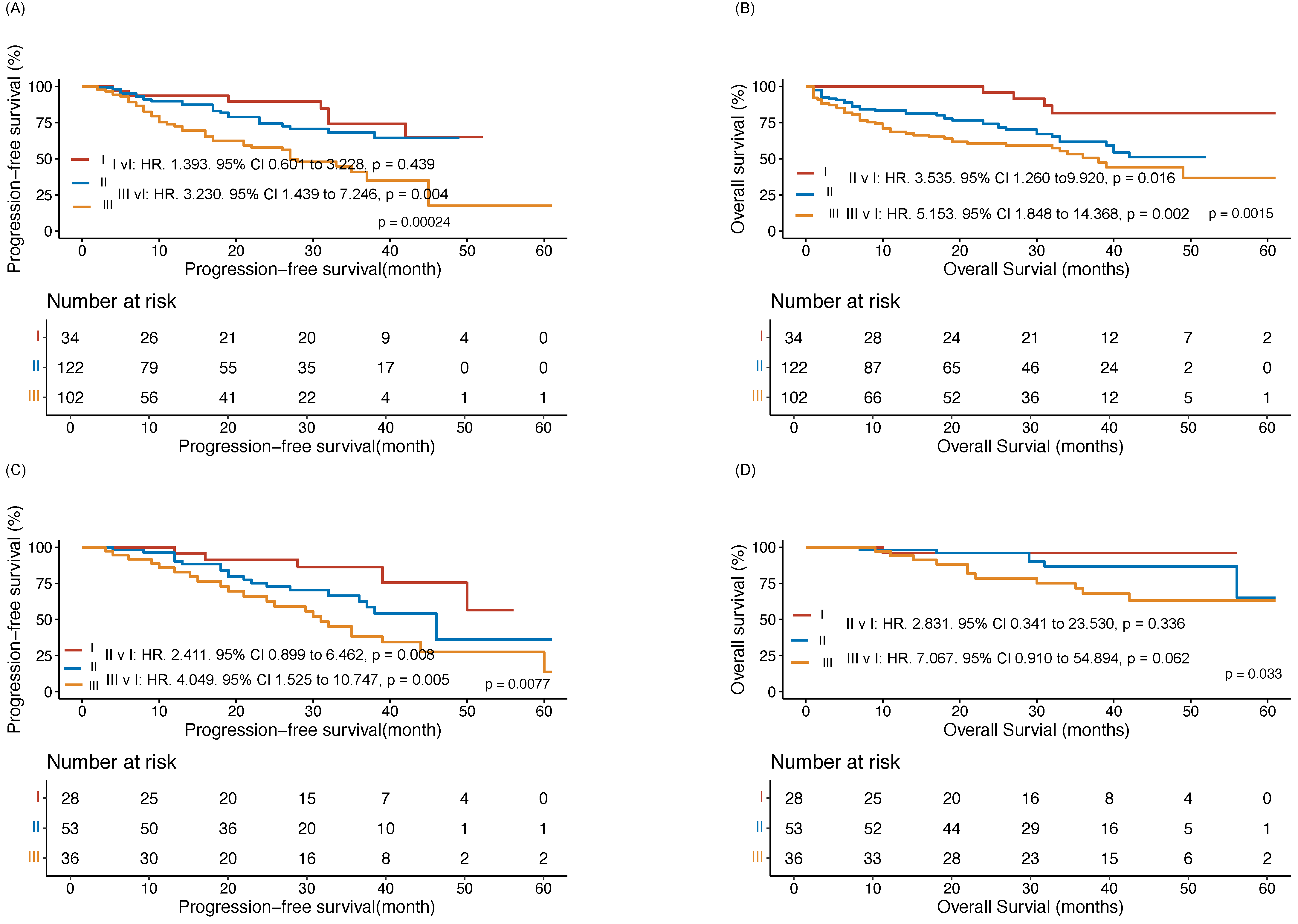


**Figure S4:**  Predictive performances of the new prediction model by transplant status. (A) PFS and OS(B) prediction by the new prediction model in patients who didn’t received ASCT; (C) PFS and OS(D) prediction by the new prediction model in in patients who received ASCT.

Figure S5

Figure 4S. ROC curves of the predictive model for the 12 month (0.72)and 36 month(0.7) probabilities of PFS in the training cohort. (A-B); ROC curves of the predictive model for the 12 month (0.6)and 36 month(0.64) probabilities of PFS in the external validation cohort; (C-D); ROC curves of the predictive model for the 12 month (0.64)and 36 month(0.6)probabilities of OS in the training cohort(E-F); ROC curves of the predictive model for the 12 month (0.64)and 36 month(0.67) probabilities of OS in the external validation cohort(G-H).

Figure S6


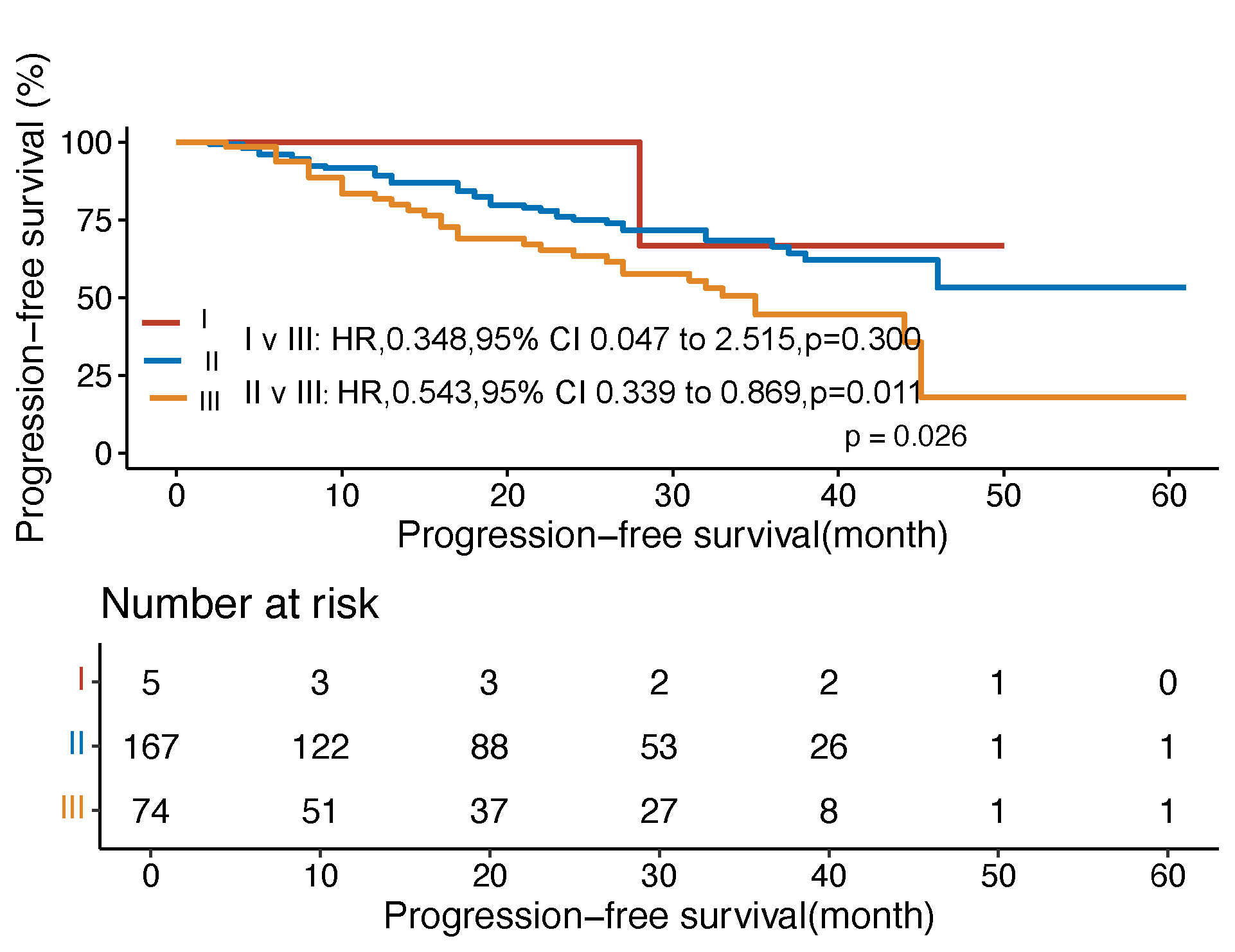


Figure S6. Adopting a new scoring system to reclassify progression-free survival (PFS) of heterogeneous R2-ISS III patients.
